# Myocardial Contractility: Historical and Contemporary Considerations

**DOI:** 10.3389/fphys.2020.00222

**Published:** 2020-03-31

**Authors:** William W. Muir, Robert L. Hamlin

**Affiliations:** ^1^College of Veterinary Medicine, Lincoln Memorial University, Harrogate, TN, United States; ^2^College of Veterinary Medicine, The Ohio State University, Columbus, OH, United States

**Keywords:** chemomechanical cycle, contractility, INOTROPY, cardiac performance, myocardial function

## Abstract

The term myocardial contractility is thought to have originated more than 125 years ago and has remained and enigma ever since. Although the term is frequently used in textbooks, editorials and contemporary manuscripts its definition remains illusive often being conflated with cardiac performance or inotropy. The absence of a universally accepted definition has led to confusion, disagreement and misconceptions among physiologists, cardiologists and safety pharmacologists regarding its definition particularly in light of new discoveries regarding the load dependent kinetics of cardiac contraction and their translation to cardiac force-velocity and ventricular pressure-volume measurements. Importantly, the Starling interpretation of force development is length-dependent while contractility is length independent. Most historical definitions employ an operational approach and define cardiac contractility in terms of the hearts mechanical properties independent of loading conditions. Literally defined the term contract infers that something has become smaller, shrunk or shortened. The addition of the suffix “ility” implies the quality of this process. The discovery and clinical investigation of small molecules that bind to sarcomeric proteins independently altering force or velocity requires that a modern definition of the term myocardial contractility be developed if the term is to persist. This review reconsiders the historical and contemporary interpretations of the terms cardiac performance and inotropy and recommends a modern definition of myocardial contractility as the preload, afterload and length-independent intrinsic kinetically controlled, chemo-mechanical processes responsible for the development of force and velocity.

## Introduction

**FIGURE 1:**
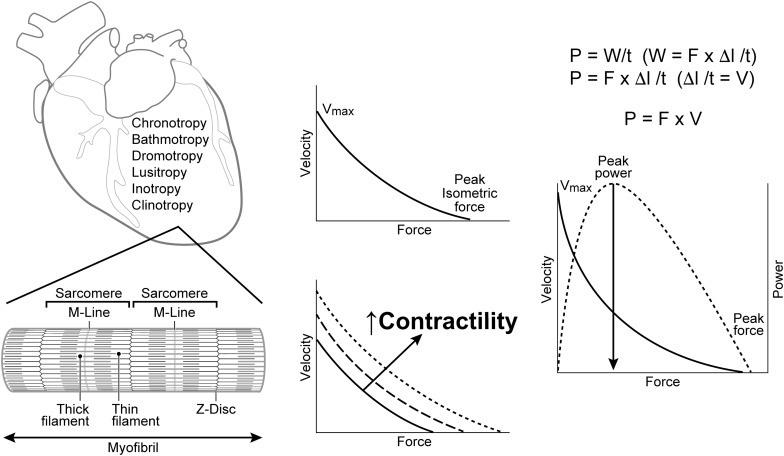
Terms used to describe the properties of cardiac cells include: chronotropy, bathmotropy, dromotropy, lusitropy, inotropy, and clinotropy. Left: Cardiac muscle fibers are comprised of repeating units of sarcomeres that are separated by *Z*-disks and contain the contractile proteins actin (thin filament) and myosin (thick filament). Center: Increases in cardiac contractility are usually represented by directionally similar changes in both force and velocity. Right: A power curve is generated as the product of force and velocity at each point along the F-V curve. V = Velocity; V_*max*_ = maximal velocity at no load; F = Force; P = power; W = work; l = length; t = time; Δ = change; M-Line = attachment site for the thick filaments and center of the sarcomere; *Z*-Disk = the anchoring point for thin filaments that separate sarcomeres.

Understanding the significance and varied uses of terms that describe cardiac function requires familiarity with the methods and limitations inherent in conducting *in vitro* and *in vivo* muscle experiments and their translation to clinical practice (Hinken and Solaro, 2007; Silverman, 2007; Sivaramakrishnan et al., 2009; Miller et al., 2010; Milani-Nejad and Janssen, 2014; Ortega et al., 2015; Sequeira and van der Velden, 2015; Spinale, 2015; Sung et al., 2015; MacLeod, 2016; Noble, 2017; Sweeney and Hammers, 2018; Sweeney and Holzbaur, 2018). Muscle contraction enables animals to move and hollow organs with one-way valves, like the heart, to generate force and transfer blood from veins to arteries. The properties of the heart that permit its function are described in terms possessing the suffix “tropic” (i.e., affecting or influenced by): chronotropic, bathmotropic, dromotropic, inotropic, lusitropic, and occasionally clinotropic (Figure 1; Spinale, 2015; Mannhardt et al., 2017; Sweeney and Holzbaur, 2018). Of these, the adjective inotropic (i.e., affecting force) or abstract noun inotropy stand out as the primary focus of more than 15,000 PubMed citations (PubMed/MEDLINE, Cochrane Library, Web of Science, ClinicalTrials.gov) that have investigated the effects of various diseases, chemical compounds, devices or toxins on the ability of the heart to develop force (positive or negative inotropes) or to improve force development in patients with various types of heart failure (HF) (Asif, 2014; Francis et al., 2014; Sohaib and Aronow, 2015; Woody et al., 2018).

Of particular relevance to the current discussion is the identification and investigation of genetic mutations and compounds that change the heart’s inotropic state by altering sarcomeric protein cross-bridge (XB) kinetics (Spudich, 2011, 2014; Ait Mou et al., 2015; Tardiff et al., 2015; Tang et al., 2017; Nanasi et al., 2018; Wang et al., 2018). The small molecule myosin regulator, omecamtiv mecarbil, for example, enhances sarcomeric force development by increasing the number and synchrony of strongly bound myosin crossbridges (XB’s), thereby increasing sarcomeric force independent of changes in intracellular calcium concentration [Ca^+2^]_*i*_, calcium transients, shortening velocity or oxygen consumption (Malik et al., 2011; Tardiff et al., 2015; Utter et al., 2015; Hashem et al., 2017; Planelles-Herrero et al., 2017; Swenson et al., 2017; Kaplinsky and Mallarkey, 2018; Spudich, 2019; Kieu et al., 2019). Alternatively, compounds that reduce sarcomeric force without changing shortening velocity or the rate of myocardial relaxation (ex. para-nitroblebbistatin; mavacapten) are being studied as treatment for hypertrophic cardiomyopathy (HCM) (Kawas et al., 2017; Tang et al., 2017; Kampourakis et al., 2018; Spudich, 2019).

Enter the ubiquitous term “contractility,” a term freely applied to describe the heart’s performance, or used as a synonym for inotropy. This conflation of terms creates misunderstandings and more importantly confuses descriptions of experimental results and test article effects.

Jewell summarized “the essence” of how muscle length regulates contraction more than 40 years ago by stating: “The end result of excitation-contraction coupling is the formation of tension-generating cross-bridges between overlapping parts of the thick (myosin) and thin (actin) filaments that make up the contractile system” (Jewell, 1977). Subsequent research has provided excellent descriptions of cardiac muscle cell electrophysiology, [Ca^+2^]_*i*_ cycling (calcium induced- calcium release) and the sequence of processes responsible for cardiac muscle contraction (excitation-contraction coupling) (Bers, 2008; Janssen, 2010; Chung et al., 2015; Sequeira and van der Velden, 2015; Eisner et al., 2017; Sweeney and Hammers, 2018). Current investigations of cardiac muscle contraction are focused on the biochemistry (i.e., chemo-mechanical cycle), mechano-sensing and kinetic behavior of sarcomeric proteins since it is generally believed that cardiac muscle contraction: “has its roots in the individual molecular motors working in every muscle cell – the myosin molecule” (Hinken and Solaro, 2007; Janssen, 2010, 2019; Sela et al., 2010; Stehle and Iorga, 2010; Spudich, 2011; Sung et al., 2015; Liu et al., 2018; Mamidi et al., 2019). This perspective provides historical and contemporary evidence for conveying definitions of the terms cardiac performance, inotropy and contractility.

## Cardiac Function: Definitions of Cardiac Performance, Inotropy and Contractility

### Cardiac Performance

Cardiac performance is the ability of the heart to pump blood into arteries and is expressed as cardiac output per unit time or as stroke volume per heartbeat. Factors that modulate the heart’s ability to pump blood (i.e., perform) include heart rate (Bristow et al., 1963; Ceconi et al., 2011), loading conditions (i.e., preload, afterload) (Milnor, 1975; Norton, 2001; Skrzypiec-Spring et al., 2007; Milan et al., 2011; Westerhof and Westerhof, 2013; O’Rourke et al., 2016), the myosin molecules contractile state (Spudich, 2011), ventricular geometry (Lieb et al., 2014), elastance (i.e., stiffness) (Fry et al., 1964; Gaasch et al., 1976; Suga et al., 1980; Sagawa, 1981; Suga, 1990; Palladino et al., 1998; Zhong et al., 2005; Campbell et al., 2008; Walley, 2016; Kerkhof et al., 2018), ventricular-vascular coupling (Kass and Kelly, 1992; Antonini-Canterin et al., 2013; Walley, 2016) and prevailing neurohumoral activity, especially sympathetic-parasympathetic tone (Thomas, 2011; Gordan et al., 2015). Changes in preload and afterload have been described as “preload reserve” and “afterload matching,” respectively (Brutsaert and Sonnenblick, 1973; Ross et al., 1976; Ross, 1983; Walley, 2016; Schotola et al., 2017; Boudoulas et al., 2018). Key determinants of pump performance include heart rate, preload (volume of blood within a chamber), afterload (hindrance to ejection), and contractility. Pump performance may decrease (e.g., decrease in heart rate or preload; increase in afterload) or increase (e.g., increase in heart rate or preload; decrease in afterload) dependent upon changes in loading conditions but independent of contractility. Cardiac performance should be operationally defined as the heart’s ability to pump blood [ex. cardiac output (CO)].

### Inotropy

The term “inotropic” is formed from the Greek word *ino* (sinew) and the suffix “*tropic*.” It is commonly employed in physiology to mean the force of muscular contraction. This usage most likely evolved from the Germanic translation of the term sinew to mean vigor, strength, or power. The terms inotropy, inotropism, inotropic, and inotrope have all been applied to describe the force or tension developed during muscle contraction. Most physiologists agree that the force generated by a contracting isolated muscle is dependent upon sarcomere length, [Ca^+2^]_*i*_, the velocity of sarcomere shortening when shortening against zero load, the type of myosin (α vs. β), and the state of phosphorylation of myosin (Sonnenblick, 1965a; Daniels et al., 1984; ter Keurs and de Tombe, 1993; Landesberg and Sideman, 2000; de Tombe and Ter Keurs, 2012; ter Keurs, 2012; Greenberg et al., 2014). Experimentally derived length-force, length-velocity, force-velocity, and length-force-velocity relationships obtained from isolated (*in vitro*) cardiac muscle fiber experiments have inextricably linked force with velocity (Sonnenblick, 1965a, b; Noble et al., 1969; Sonnenblick et al., 1969; Hugenholtz et al., 1970; Gulch and Jacob, 1975; Wikman-Coffelt et al., 1982; ter Keurs and de Tombe, 1993; de Tombe and Ter Keurs, 2012).

Inotropy is muscle fiber length dependent and is modified by heterometric autoregulation [Cyon-Frank-Starling mechanism (Zimmer, 1998, 2002; Katz, 2002; Amiad and Landesberg, 2016; Sequeira and van der Velden, 2017)], homeometric autoregulation [von Anrep effect *in vivo*; slow force response *in vitro* (Sarnoff et al., 1960; Cingolani et al., 2013; Clancy et al., 1968; Furst, 2015; Schotola et al., 2017)], the force-frequency relationship [Bowditch, treppe, staircase effect, chronotropic-inotropy (Bowditch, 1871; Noble et al., 1966; Anderson et al., 1973; Higgins et al., 1973; Gwathmey et al., 1990; Ross et al., 1995; Endoh, 2004; Janssen and Periasamy, 2007; Janssen, 2010; Puglisi et al., 2013)], and autonomic activity (Glick and Braunwald, 1965; Thames and Kontos, 1970; Ross et al., 1995). Inotropy decreases almost instantly, within one heartbeat, when parasympathetic activity increases, and more slowly, over 6–8 s, when sympathetic efferent activity changes (Olshansky et al., 2008). The term inotropy and its derivatives should be operationally defined as force.

### Contractility

The term “contractility” is historically embedded in both the experimental and clinical cardiovascular literature and is formed from the adjective *contractile* and the suffix “*ility*” (i.e., quality), thereby forming the abstract noun contractility. The word contractile (derived from French: 1706) implies that something has the ability to shrink or shorten. Striated muscle cells and sarcomeres shrink because the contractile proteins (i.e., actin, myosin) slide past one another, developing a force that pulls the sarcomeric Z-bands closer together. The proximate source of contraction, however, arises from the cycling of actomyosin XBs that apply force to actin filaments (Sequeira and van der Velden, 2015: Sweeney and Holzbaur, 2018).

One editor in chief of a highly respected research journal has stated that, “the term contractility remains useful in order to permit succinct written and oral communication between and among scientists and clinicians” (Solaro, 2011). Others are not convinced and consider the term to be “hotly debated” (Sweeney and Hammers, 2018) and “ill-defined.”^1^ Many authors equate the term with inotropy (Anderson et al., 1973; Noble, 1973; Solaro, 2011; Spinale, 2015). One noted cardiovascular scholar went so far as to state, “If it weren’t for pressure-volume (P-V) loops and the end systolic pressure volume relationship (ESPVR), the term contractility would likely have disappeared” (Mirsky, 1974). The same author went on to state, “In view of the vagueness of the definitions, it may be worthwhile in the future to eliminate this term (i.e., contractility) entirely from the literature” (Mirsky, 1974). Shepherd and Vanhoutte proposed that cardiac contractility be defined as, “the intensity of the active state” that is evoked, “by the interaction between the actin and myosin molecules as a consequence of the integration of the biochemical and biophysical events resulting in the actomyosin interaction described only by a complex relationship between the force exerted by the muscle, the velocity of shortening, its length and the time of the contractile cycle at which these parameters are measured” (Shepherd and Venhoutte, 1979). Other noted muscle physiologists and clinical cardiologists have provided more detailed definitions by asserting that contractility implies the intrinsic ability of the heart, at a fixed heart rate, to generate force and shorten independent of preload and afterload (Noble, 1973; Strobeck and Sonnenblick, 1981; Burkhoff et al., 1987; Abraham et al., 2003; Bombardini, 2005). A focused group of molecular physiologists stated that, “Cardiac contractility can be defined as the tension developed and velocity of shortening (i.e., the “strength” of contraction) of myocardial fibers at a given preload and afterload. It represents a unique and intrinsic ability of cardiac muscle (contracting at a fixed heart rate) to generate a force that is independent of any load or stretch applied” (Wijayasiri et al., 2012). Integral to all current definitions of the term contractility is the tacit requirement that it is independent of loading conditions (Braunwald, 1971; Davidson et al., 1974; Katz, 1983; Kass et al., 1987; Penefsky, 1994; Bombardini, 2005; Schotola, et al., 2017) (See footenote 1).

Much of what is known about the contractile properties of the heart has been derived from *in vitro* experiments conducted on skeletal or cardiac muscle strips, fascicles, or fibers (Sonnenblick, 1965a; Katz, 1983; Gibbs, 1987; Katz, 2002: Batters et al., 2014) obtained from various mammalian species (Abbott and Mommaerts, 1959; Jewell, 1977; Sequeira and van der Velden, 2015; de Tombe and Ter Keurs, 2016; Sweeney and Hammers, 2018) in solutions where the [Ca^+2^]_*i*_ was manipulated (Noble et al., 1969; Kentish and Stienen, 1994; Noble, 2017). Pairs of myosin heads encircle the thick myosin filament backbone in a helical or quasi-helical fashion and are described as existing in one of three transitional states: active, or when not active, disordered relaxed (DRX; approximately 50–60%) and super-relaxed (SRX: approximately 40–50%) (McNamara et al., 2015; Anderson et al., 2018). Only 10–30% of the total myosin S1 heads develop strong bonds and complete one or two XB cycles during the contractile period (low duty ratio) (Spudich, 2011; Batters et al., 2014). Various mechanical properties have been deduced from stretched (i.e., preloaded) cardiac muscle fibers stimulated to contract against varying (i.e., isotonic) or an infinite (i.e., isometric) load (i.e., afterload) (Sonnenblick, 1965a; Sonnenblick, 1965b; Noble et al., 1969; Daniels et al., 1984; McDonald, 2011). Notably, the tissues visco-elastic properties permit the sarcomeres (i.e., contractile units) in each muscle fiber to shorten and develop force (i.e., tension) regardless of the imposed loading conditions, whether or not the muscle fiber shortens (Gaasch et al., 1976; Suga, 1990; de Tombe and Ter Keurs, 2016). The force-velocity relation extrapolated to zero load (i.e., V_*max*_) has long been considered a load independent and “complete” (Sonnenblick et al., 1969) measure of cardiac contractility, particularly in isolated muscle strips (Ross et al., 1966; Sonnenblick et al., 1969; Hugenholtz et al., 1970; Mason et al., 1971; Parmley et al., 1972; Brutsaert and Sonnenblick, 1973; Kettunen et al., 1986) so long as it is “reflected by maximum force development as well as the velocity of shortening” (Strobeck and Sonnenblick, 1981). These studies have determined that muscle shortening velocity is inversely related to force generation; an increase in muscle (i.e., sarcomere) length, at any given load, increases shortening velocity; force and velocity development are tightly correlated with ATP utilization and oxygen consumption; and the maximal velocity of sarcomere shortening is, in part, dependent upon myosin composition and the degree of synchrony among the less than 30% of XBs that cycle during each resting cardiac contraction (Barany, 1967; Graham et al., 1968; Daniels et al., 1984; Sieck and Regnier, 1985; de Tombe and Ter Keurs, 2016).

Contemporary experimental studies of muscle contraction suggest that, “There is an apparent gap between basic and clinical science methods and measurements investigating cardiac contractile function, making it difficult to directly relate specific parameters of a XB cycle to the events in a cardiac cycle. But it is clear that ventricular contractile function is heavily dependent on XB kinetics” (Mamidi et al., 2018). Kinetic and cooperative load-dependent processes have determined that the myosin-actin attachment rate, overall cycle rate, the amount of time that myosin is attached to actin, and the total number of myosin heads in the active state (i.e., duty ratio) are all determinants of force (F) development, sliding velocity (V_*max*_) and the amount of ATP consumed (Stehle and Iorga, 2010; Swenson et al., 2017; Liu et al., 2018; Sweeney and Holzbaur, 2018; Janssen, 2019). Harmonic force spectroscopy (HFS) experiments have provided further insights into how myosin’s length-dependent kinetics (detachment: k_*det*_; recruitment: k_*rec*_) control XB transitions and are modified by biologic alterations that include small regulatory molecule conversions (Piazzesi et al., 1997; Stelzer et al., 2006; Ford et al., 2010; Sung et al., 2015; Janssen, 2019). Myosin head detachment rate (i.e., k_*det*_) has been identified as a key parameter influencing contractility because it determines the time myosin is bound to actin in a force producing state (Janssen, 2010; Greenberg et al., 2014; Sung et al., 2015; Liu et al., 2018). The discovery of a host of cardiomyopathy mutations and a new generation of chemical compounds that modify myosin “motor” kinetics and chemo-mechanical processes (Malik et al., 2011; Spudich, 2014; Tardiff et al., 2015; Nanasi et al., 2016, 2018; Swenson et al., 2017; Kampourakis et al., 2018; Mamidi et al., 2018), which produce divergent effects on force and velocity suggest that revival of the term clinotropy (i.e., velocity) should be considered in order to more holistically define the term contractility as force (i.e., intoropy) and velocity (i.e., clinitropy) (MacLeod, 2016). Myocardial contractility should be defined as the load and length-independent, kinetically controlled, chemo-mechanical processes responsible for the development of force (inotropy) and velocity (clinotropy).

## The Difference Between Cardiac Performance, Inotropy and Contractility in Normal and Diseased Hearts

Cardiac contractility should not be confused or conflated with cardiac performance (Braunwald, 1971; Ross, 1983; Sequeira and van der Velden, 2015). The primary functions of the ventricles are to pump blood (vis a tergo) and to draw blood (vis a fronte) from the atria (Chung et al., 2015; LaGerche and Claus, 2015; Wang et al., 2018). The ability of the heart to perform this task (i.e., create blood flow) is dependent upon heart rate and loading conditions (i.e., preload, afterload) and by how well and at what cost (i.e., O_2_ consumption) the left and right ventricles (LV and RV, respectively) produce pressure gradients.

Cardiac pump performance can deteriorate, improve or remain the same when cardiac contractility is normal or abnormal, depending upon the magnitude of changes in heart rate and loading conditions (i.e., preload; afterload) (Wiggers, 1951; Ross et al., 1976; Ross, 1983). This conclusion is highlighted by clinical scenarios wherein the heart’s pumping performance is enhanced by compensatory mechanisms (e.g., increased heart rate or preload, neuro-humeral activation) or drugs (e.g., antiarrhythmics, vasodilators), which improve cardiac output when cardiac contractility is decreased, or conversely, decrease cardiac output (i.e., increased afterload, blood loss, valvular stenosis or incompetence) when cardiac contractility is normal or enhanced (Triposkiadis et al., 2009; Florea and Cohn, 2014; Tint et al., 2019). For example, aortic or mitral valve insufficiency can reduce cardiac output when cardiac contractility is increased (Chatterjee and Parmley, 1977; Ross, 1983; Kittleson et al., 1984).

Myocardial contractility is not only the ability of the heart to develop force (i.e., inotropy) but also the ability to generate velocity (Sonnenblick, 1965a, b; Sonnenblick et al., 1969; Mason et al., 1971; de Tombe and Ter Keurs, 2016; Liu et al., 2018; Janssen, 2019). Genetic mutations and drugs that alter the cardiac actin-activated chemo-mechanical ATPase cycle and XB kinetics (i.e., k_*det*_) may cause force and velocity to change independently (Anderson et al., 2018; Liu et al., 2018; Nanasi et al., 2018).

## Conclusion

Cardiac muscle contraction “has its roots in the individual molecular motors working in every muscle cell – the myosin molecule” (Liu et al., 2018). Myocardial contraction is mechanically manifested as the force and velocity generated during sarcomere shortening. It occurs when Ca^+2^ binds to troponin-C and reconfigures tropomyosin so that myosin heads fueled by the energy produced from ATP hydrolysis produce effective XB cycling.

The question of acceptability or usefulness of the term cardiac contractility should be based upon whether the word, contractility, can actually define a unique physiological process that is quantifiable. A multitude of methods have evolved for quantifying myocardial contractility, all of which are dependent upon the ability of sarcomeres to develop force and velocity (Urschel et al., 1980; Naiyanetr, 2013; Spinale, 2015; Kohli and Kovacs, 2017). Myocardial contractility should be defined as the load and length-independent, intrinsic, kinetically controlled, chemo-mechanical processes responsible for the development of force (inotropy) and velocity (clinotropy).

## Author Contributions

WM reviewed the literature, organized and drafted the work and designed the figure. RH drafted and contributed key components to the discussion of inotropy.

## Conflict of Interest

The authors declare that the research was conducted in the absence of any commercial or financial relationships that could be construed as a potential conflict of interest.

## Footnotes

1 Burkhoff D: http://www.columbia.edu/itc/gsas/g6001/BasicLVMechanics.pdf
